# Genetic and environmental risk factors of acute infection-triggered encephalopathy

**DOI:** 10.3389/fnins.2023.1119708

**Published:** 2023-01-24

**Authors:** Masashi Mizuguchi, Akiko Shibata, Mariko Kasai, Ai Hoshino

**Affiliations:** ^1^Department of Developmental Medical Sciences, Graduate School of Medicine, The University of Tokyo, Tokyo, Japan; ^2^Department of Pediatrics, National Rehabilitation Center for Children With Disabilities, Tokyo, Japan; ^3^Laboratory for Brain Development and Disorders, RIKEN Center for Brain Science, Tokyo, Japan; ^4^Department of Pediatrics, Saitama Citizens Medical Center, Saitama, Japan; ^5^Department of Neuropediatrics, Tokyo Metropolitan Neurological Hospital, Fuchu, Japan

**Keywords:** acute encephalopathy, infection, drug, immune response, metabolism, neuronal excitation, susceptibility gene

## Abstract

Acute encephalopathy is a constellation of syndromes in which immune response, metabolism and neuronal excitation are affected in a variable fashion. Most of the syndromes are complex disorders, caused or aggravated by multiple, genetic and environmental risk factors. Environmental factors include pathogenic microorganisms of the antecedent infection such as influenza virus, human herpesvirus-6 and enterohemorrhagic *Escherichia coli*, and drugs such as non-steroidal anti-inflammatory drugs, valproate and theophylline. Genetic factors include mutations such as rare variants of the *SCN1A* and *RANBP2* genes, and polymorphisms such as thermolabile *CPT2* variants and *HLA* genotypes. By altering immune response, metabolism or neuronal excitation, these factors complicate the pathologic process. On the other hand, some of them could provide promising targets to prevent or treat acute encephalopathy.

## Introduction

Acute encephalopathy is a severe brain complication of infection, characterized clinically by acute onset of severe and long-lasting disturbance of consciousness, usually accompanied by seizures. The pathologic substrate of acute encephalopathy is diffuse or widespread, non-inflammatory brain edema, which can be visualized by neuroimaging techniques such as cranial magnetic resonance imaging (MRI) and computed tomography (CT). Acute encephalopathy consists of multiple syndromes, between which there are both similarities and differences (Mizuguchi et al., 2007). Acute encephalopathy may occur at any age but is most common in infancy and childhood. The onset is usually preceded by common infectious diseases, mostly febrile, such as influenza, exanthem subitum and rotavirus gastroenteritis. The incidence of each syndrome is variable to a great extent among countries and ethnicities.

Previous studies have provided numerous pieces of information on the involvement of many risk factors in the etiology and pathogenesis of acute encephalopathy. Environmental factors include pathogens of the antecedent infection, drugs and toxins, whereas genetic factors include gene mutations and polymorphisms. The aim of this review is to show a comprehensive list of the factors, and to describe how they cause or aggravate brain edema to cause acute encephalopathy.

## Acute encephalopathy syndromes

**FIGURE 1:**
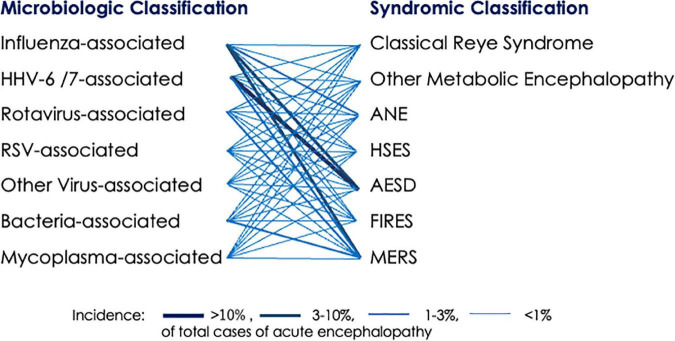
Association between pathogens of antecedent infection and encephalopathy syndromes. A bold line indicates high incidence, based on epidemiologic data in Japan (Hoshino et al., 2012; Kasai et al., 2020). ANE, acute necrotizing encephalopathy; AESD, acute encephalopathy with biphasic seizures and late reduced diffusion; FIRES, febrile infection-related epilepsy syndrome; HHV, human herpesvirus; HSES, hemorrhagic shock and encephalopathy syndrome; MERS, clinically mild encephalitis/encephalopathy with reversible splenial lesion; RSV, respiratory syncytial virus.

Acute encephalopathy is classified in two ways: microbiologic classification based on the pathogen of antecedent infection, such as influenza-associated encephalopathy, human herpesvirus-6 (HHV-6)-associated encephalopathy, rotavirus-associated encephalopathy and severe acute respiratory syndrome coronavirus-2 (SARS CoV-2)-associated encephalopathy, and syndromic classification based on the clinical and neuroimaging features (Table 1), such as acute necrotizing encephalopathy (ANE), acute encephalopathy with biphasic seizures and late reduced diffusion (AESD), clinically mild encephalitis/encephalopathy with a reversible splenial lesion (MERS) and febrile infection-related epilepsy syndrome (FIRES, also known as acute encephalitis with refractory, repetitive partial seizures) (Mizuguchi et al., 2007; Mizuguchi et al., 2021; Figure 1). Among these syndromes, prognosis varies to a large extent. For example, ANE is characterized by a high fatality (26–28%) and a high rate of neurologic sequelae (45–56%), AESD by a low fatality (1–2%) and a high rate of neurologic sequelae (61–66%), and MERS by a low fatality (0%) and a low rate of neurologic sequelae (5–7%) (Hoshino et al., 2012; Kasai et al., 2020).

**TABLE 1:** Major acute encephalopathy syndromes: Pathogenesis, clinical features and risk factors.

Syndrome	Classical reye syndrome	Acute necrotizing encephalopathy (ANE)	Acute encephalopathy with biphasic seizures and late reduced diffusion (AESD)	Clinically mild encephalitis/encephalopathy with a reversible splenial lesion (MERS)
Suspected pathogenesis	Metabolic error: mitochondrial dysfunction	Dysregulated inflammation: cytokine storm	Excessive neural excitation: excitotoxicity	Loss of myelin integration
Main clinical features	Hepatic dysfunction Hypoglycemia Hyperammonemia	Vascular brain edema (thalamic) MOF, DIC	Cytotoxic brain edema (subcortical) Status epilepticus Biphasic clinical course	Intra-myelin edema Mild and transient brain dysfunction
Genetic susceptibility: mutations	Genes encoding metabolic enzymes	*RANBP2*	*SCN1A*, *SCN2A HNPRU* Others	*MYRF*
Genetic susceptibility: polymorphisms	Genes encoding metabolic enzymes	*IL10* HLA-DR and -DQ	*ADORA2A* *IL1B* *STK39* *CPT2* HLA-DP	*CPT2*
Environmental risk factors: infections	Influenza and other viruses	Influenza and other viruses	HHV-6/7 and other viruses	Influenza and other viruses Bacteria
Environmental risk factors: drugs	Aspirin Valproate Pivalate-containing antibiotics	NSAIDs	Theophylline	

### Pathogenesis of acute encephalopathy

**FIGURE 2:**
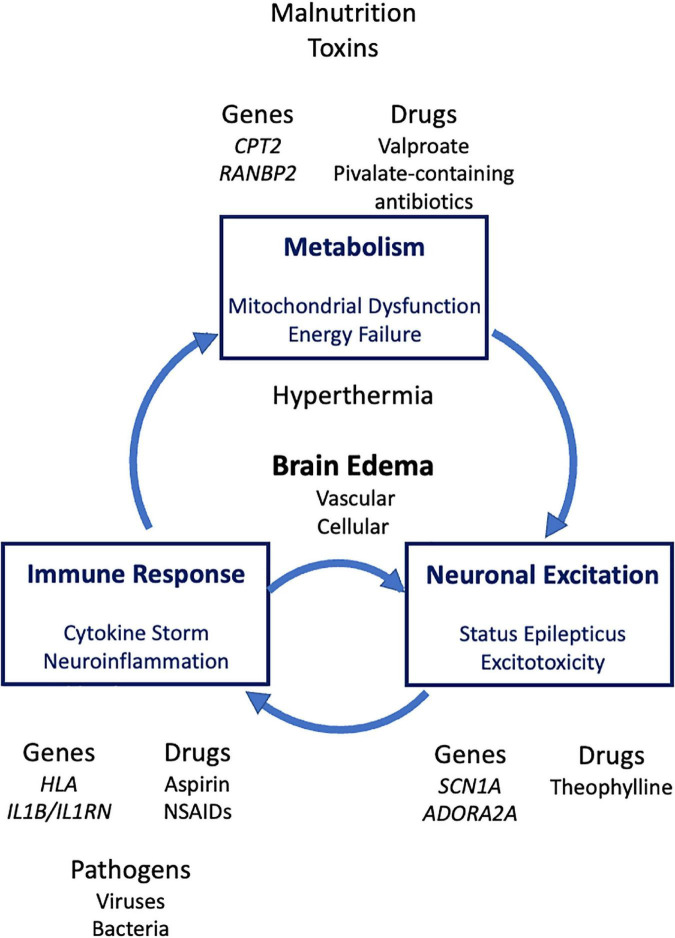
Genetic and environmental risk factors and major pathologic processes of severe acute encephalopathy. HLA, human leukocyte antigen; NSAIDs, non-steroidal anti-inflammatory drugs.

In severe syndromes, there are three major pathogenetic events: dysregulated immune responses, defective energy metabolism and excessive neuronal excitation (Figure 2). Clinical and laboratory findings of ANE include signs of systemic inflammatory response syndrome such as multiple organ failure (MOF) and disseminated intravascular coagulation (DIC) (Mizuguchi et al., 2007), as well as very high levels of inflammatory cytokines in the serum (Ichiyama et al., 2003a,b), whereas neuroimaging findings characteristic of ANE are symmetric brain lesions in the bilateral thalamus, showing hemorrhage in the center and vasogenic edema in the periphery (Mizuguchi et al., 2002). Based on these findings, the main pathomechanism of ANE is considered to be dysregulated immune responses, or cytokine storm in which the brain is damaged as a primary target organ.

Biochemical findings of classical Reye syndrome include hyperammonemia, hypoglycemia and free fatty acidemia. Previous studies, mostly in the 20th century, have proven this syndrome as a transient disorder of mitochondria that regulate urea cycle, gluconeogenesis and fatty acid oxidation (Visentin et al., 1995).

Clinical picture of AESD is characterized by biphasic course consisting of acute stage (within 1 week after onset) and subacute stage (1 week–1 month after onset), and by delayed appearance of cerebral lesions representing cytotoxic edema in the subcortical white matter (Takanashi et al., 2006). Neuronal apoptosis is suggested by the serial change of cytochrome *c* in the cerebrospinal fluid (Hosoya et al., 2005), and excitotoxicity by an increase in glutamate in the subcortical lesion demonstrated by magnetic resonance spectroscopy (Takanashi et al., 2009; Takanashi et al., 2015). Thus, AESD is considered to be caused by excessive neuronal excitation.

The three pathogenetic processes are mutually related. First, in classical Reye syndrome, proinflammatory cytokines, such as tumor necrosis factor (TNF), mediate the metabolic effects of toxins and drugs causative of this syndrome (Larrick and Kunkel, 1986), indicating that cytokine storm impairs mitochondrial function. Second, mitochondrial encephalopathy syndromes, such as Leigh syndrome and mitochondrial myopathy, encephalopathy, lactic acidosis and stroke-like episodes (MELAS), are usually manifested with severe epileptic seizures, since neurons are the most energy-consuming cell type vulnerable to energy failure due to mitochondrial dysfunction. Third, severe epileptic seizures or status epilepticus induces proinflammatory responses in the brain (Vezzani and Granata, 2005; Vezzani et al., 2008), which in turn affect blood-brain barrier (Oby and Janigro, 2006). Conversely, proinflammatory molecules such as interleukin 1β (IL-1β) and TNF increase neuronal excitability (Nabbout et al., 2011). IL-1β plays a major role in the precipitation of seizures associated with fever (Heida et al., 2009). Taken together, inflammation is both a cause and consequence of seizures. Dysregulated immune response and excessive neuronal excitation form a “small” vicious cycle that further fosters neuroinflammation and status epilepticus (Nabbout et al., 2011; Figure 2).

Based on these relationships, the three pathogenetic changes, inflammatory, metabolic and neuronal (epileptic), may form a “large” vicious cycle (Figure 2). Two or three of them may co-exist in very severe syndromes of acute encephalopathy. For example, hemorrhagic shock and encephalopathy syndrome shows both findings of cytokine storm such as fever, shock, DIC, and MOF (Rinka et al., 2008), and those of neuronal over-excitation such as “electrical storms” on electroencephalogram (EEG), a severe form of subclinical status epilepticus (Harden et al., 1991), and “bright tree appearance” on magnetic resonance imaging, cerebral subcortical lesions of reduced diffusion mimicking those of AESD (Kuki et al., 2021).

### Acute encephalopathy as a complex disorder

The onset and evolution of pathologic process in acute encephalopathy involve multiple initiating and aggravating factors, some of which are environmental. Pathogens of antecedent infections, typically with high fever, are an essential trigger. Fasting (malnutrition) and/or excessive protein intake may also trigger metabolic encephalopathies including classical Reye syndrome. Drugs and toxins may worsen acute encephalopathy. For example, aspirin and aflatoxin play major roles in the pathogenesis of classical Reye syndrome (De Vivo, 1985).

The involvement of genetic factors is suggested by the uneven geographic distribution of cases. As described below, the commonest pathogen of acute encephalopathy are common viruses such as influenza virus, HHV-6/7 and rotavirus, all of which are distributed worldwide. On the other hand, the incidence of various encephalopathy syndromes is highly variable among countries and ethnicities. For example, AESD is the most common syndrome in Japan (Hoshino et al., 2012; Kasai et al., 2020), but is rarely reported from other countries. The number of sporadic cases of ANE is much larger in east Asian countries than in west Asia, Europe, and America, whereas that of familial/recurrent ANE (ANE1) is much smaller. These geographic and ethnic differences, together with the presence of rare familial cases, suggest genetic susceptibility to acute encephalopathy.

When the total sum of effects of these genetic and environmental factors exceeds a certain threshold, acute encephalopathy may occur. This notion applies even to an encephalopathy syndrome showing Mendelian inheritance, given the low penetrance (40–50%) of ANE1 (an autosomal dominantly inherited disorder caused by a gene mutation) in which the involvement of other genetic (thermolabile variants of a mitochondrial enzyme) and environmental factors (influenza virus) are described (Neilson et al., 2003; Neilson et al., 2004; Ohashi et al., 2021).

## Infectious agents

### Viral infections

In the majority of acute encephalopathy cases, the antecedent infection is caused by common viruses that often affect young children to produce high fever. According to a Japanese survey on the epidemiology of acute encephalopathy conducted twice (the first in 2010 and second in 2017), the two most common viruses were influenza virus and HHV-6/7, followed by rotavirus and respiratory syncytial virus (RSV) (Hoshino et al., 2012; Kasai et al., 2020).

Association of viruses with syndromes is non-specific, and any virus can cause any syndrome. For example, clinical manifestation of ANE shows no difference between influenza virus and other viruses (Okumura et al., 2009). However, the Japanese survey has noted strong epidemiologic associations between exanthem subitum (HHV-6/7) and AESD, between influenza and ANE, between influenza and MERS, and between rotavirus gastroenteritis and MERS (Figure 1; Hoshino et al., 2012). The reason for these associations remains obscure, but there are two possible explanations. One is that different viruses elicit host immune responses with distinct cytokine profiles, thereby triggering certain syndromes of acute encephalopathy. Another is the relationship between the age predilection of a viral infection and age-dependent vulnerability of developing brain to specific types of insults, based on the degree of brain maturation.

Influenza virus is the commonest causative pathogen of acute encephalopathy. In Japan, age distribution of influenza encephalopathy shows its peak at 1 year, and median at 6 years, suggesting that the risk of severe brain complication (acute encephalopathy) is higher in primary infection than in re-infections. Males and females are equally affected. The seasonality of influenza-associated encephalopathy is the same as that of influenza. The incidence of encephalopathy tends to be higher in A(H3N2) epidemic than in A(H1N1) epidemic (Mizuguchi et al., 2007). In 2009, however, pandemic A(H1N1) was associated with a remarkable increase in cases of influenza-associated encephalopathy (Kasai et al., 2020). With regard to syndromes, influenza is the commonest pathogen in MERS and ANE (Hoshino et al., 2012).

Human herpesvirus-6/7 is the second commonest pathogen of acute encephalopathy. Age distribution shows a sharp peak at 1 year of age. Males and females are equally affected. Seasonal and yearly fluctuation of incidence is small. AESD is by far the commonest syndrome of HHV-6/7 encephalopathy, whereas HHV-6/7 is the commonest pathogen in AESD (Hoshino et al., 2012).

Severe acute respiratory syndrome coronavirus-2 has recently been added to the list of pathogenic viruses of antecedent infections. COVID-19 is occasionally complicated by severe acute encephalopathy, including ANE, hemorrhagic shock, and encephalopathy syndrome, and encephalopathy with fulminant cerebral edema (LaRovere et al., 2021; Ray et al., 2021). COVID-19-associated multiple inflammatory syndrome in children (MIS-C) or pediatric inflammatory syndrome (PIMS) is often complicated by acute encephalopathy with lesions in the splenium of corpus callosum characteristic of MERS (Abdel-Mannan et al., 2020; Lindan et al., 2021).

### Bacterial infections

Acute encephalopathy is a severe brain complication of bacterial gastroenteritis caused by enterohemorrhagic *Escherichia coli*, *Shigella*, *Salmonella*, *Yersinia, Campylobacter*, and *Bacillus cereus*. Clinical manifestations of the bacterial encephalopathy are largely different from those of viral encephalopathy, since toxins characteristic of each bacteria species, such as verotoxin (Shiga toxin), Salmonella toxin and endotoxins, play major roles in the pathogenesis of encephalopathy (Mizuguchi et al., 2001; Lingwood, 2020).

On the other hand, acute focal glomerular nephritis (AFGN) is often complicated by MERS, a syndrome usually associated with influenza virus, rotavirus and other viruses. In AFGN-associated MERS, increased cytokines and chemokines in the blood and cerebrospinal fluid are implicated (Kometani et al., 2014; Okada et al., 2022). According to the Japanese epidemiological survey, bacterial infections including AFGN account for 3–4% of MERS cases (Hoshino et al., 2012). By contrast, antecedent bacterial infections are very rare in cases of AESD and ANE (Yamaguchi et al., 2018; Huber et al., 2020).

## Drugs

### Drugs affecting immune responses and/or metabolism

Diclofenac sodium and mefenamic acid are non-steroidal anti-inflammatory drugs (NSAIDs) with a potent antipyretic effect. A Japanese study on the epidemiology of influenza-associated encephalopathy revealed that the use of these NSAIDs was associated with an increase in mortality (Nagao et al., 2008). Many of the fatal cases had cytokine storm, typically ANE (Ichiyama et al., 2003a). To explore the reason for this seemingly paradoxical aggravation, an *in vitro* study found an increase in nitric oxide production in astrocytes activated by proinflammatory cytokines such as IL-1β and TNF (Kakita et al., 2009). NSAIDs inhibit cyclooxygenase and induce proinflammatory cytokines, which in turn damages cerebrovascular endothelial cells, causing brain edema.

Aspirin (salicylate), an antipyretic which had widely been used for febrile infections of children until the 1970’s, may trigger or aggravate acute encephalopathy including classical Reye syndrome (Hurwitz et al., 1985). Since the 1980’s when warnings were issued about the use of aspirin, the incidence of this syndrome has sharply declined (Belay et al., 1999). In the pathogenesis of aspirin-induced encephalopathy, multiple mechanisms are involved. First, aspirin enhance the release of IL-1 and TNF from macrophage, which in turn impair metabolism in the liver (Larrick and Kunkel, 1986). Second, aspirin can directly inhibit hepatic mitochondria (Trost and Lemasters, 1997; Pessayre et al., 1999).

### Drugs affecting mitochondrial metabolism

Sodium valproate (VPA), a widely used antiepileptic drug, may elicit adverse effects to cause hepatotoxicity and encephalopathy, or classical Reye syndrome (Baganz and Dross, 1994; Verrotti et al., 2002; Segura-Bruna et al., 2006). Other risk factors of this encephalopathy include infants under 2 years of age, other antiepileptic drugs (phenytoin, phenobarbital, and topiramate), metabolic diseases (urea cycle disorders), excessive protein intake and malnutrition (Honeycutt et al., 1992; Kay et al., 1986). Hyperammonemia, a common finding in valproate-induced encephalopathy, is caused either by inhibition by valproyl CoA of carbamyl phosphate synthase-1, a key enzyme of urea cycle, or by secondary carnitine deficiency disturbing mitochondrial metabolism such as beta-oxidation (Warter et al., 1983; Kay et al., 1986; Duarte et al., 1993; Fromenty and Pessayre, 1995).

Glycerol and fructose, an osmotic agent injected intravenously to treat cerebral edema, may paradoxically cause acute encephalopathy in neonates, undernourished infants, and congenital metabolic disorders of glycerol and fructose such as fructose-1,6-bisphosphatse deficiency (Hasegawa et al., 2003).

Pivalate-containing antibiotics may cause secondary hypocarnitinemia, occasionally leading to acute encephalopathy with or without hypoglycemia (Stanley, 2004; Kobayashi et al., 2016). Other risk factors include congenital metabolic disorders, valproate treatment, blood and peritoneal dialysis, total parenteral nutrition, dietary restriction, anti-cancer agents, liver failure, neuromuscular disorders, severe motor and intellectual disabilities, anorexia and malnutrition (Lokrantz et al., 2004; Makino et al., 2007).

### Drugs affecting neuronal excitation

Theophylline, a xanthine derivative, is a non-selective, competitive antagonist of adenosine. There are in the human brain anti-excitatory adenosine A1 receptor (Fukuda et al., 2010) and pro-excitatory A2A receptor (Fukuda et al., 2011). Since the action of A1 receptor predominate over that of A2A receptor, a net effect of adenosine is anti-excitatory (an endogenous anticonvulsant) and that of theophylline pro-excitatory. In addition, theophylline inhibits pyridoxal kinase, causing vitamin B_6_ deficiency and a decrease in gamma-aminobutyric acid (GABA) (Ubbink et al., 1989). In Japan, theophylline had widely been used as a bronchodilator to treat an acute attack of bronchial asthma and “asthmatic bronchitis,” an acute respiratory infection of children with wheezing, until the 2000’s, when the Japanese Guidelines for the Treatment and Management of Bronchial Asthma of Children were revised. Triggered by fever, some Japanese children taking theophylline had convulsive seizures, which were often prolonged and refractory to intravenous benzodiazepines, the first-line anti-convulsant used in pediatric emergency rooms (Nakada et al., 1983; Yoshikawa, 2007; Korematsu et al., 2008). Super-refractory seizures often required treatment with a high-dose, intravenous barbiturate under mechanical ventilation. After several weeks of intensive care, most of the patients were left with severe neurologic handicaps including severe motor and intellectual disabilities and intractable epilepsy. Many of them were diagnosed with AESD (Saitoh et al., 2015b) and others with unclassified encephalopathy (Shiihara et al., 2006).

## Susceptibility genes

In a small number of familial cases, acute encephalopathy shows Mendelian inheritance. To date, two syndromes, ANE1 and familial/recurrent MERS (encephalopathy with reversible myelin vacuolation), have been documented to be inherited in an autosomal dominant fashion. Previous studies to explore their genetic background adopted hypothesis-free approaches: genome-wide linkage analysis followed by high-throughput sequencing for ANE1, and whole exome sequencing analysis for familial/recurrent MERS, both of which successfully discovered their causative genes (Neilson et al., 2004; Neilson et al., 2009; Kurahashi et al., 2018).

In a large number of sporadic cases, acute encephalopathy is a complex disorder in which multiple genetic factors are likely involved. Most genetic studies have adopted a case-control association study. Because of a small sample size resulting from the low incidence of acute encephalopathy, few studies have adopted hypothesis-free approaches, such as genome-wide association study (GWAS) (Kasai et al., 2022a). The vast majority of studies have used a candidate gene approach to search for common and/or rare variants (mutations and/or polymorphisms), based either on common disease-common variants hypothesis and/or common disease-multiple rare variants hypothesis (Shibata et al., 2020). In consideration of the suspected pathomechanism of acute encephalopathy described above, genes related to immune responses, metabolism and neuronal excitation have been selected as candidate genes in many of the genetic studies.

### Genes regulating immune response

Human leukocyte antigen (*HLA*) genotypes include *HLA* class I (HLA-A, -C, and -B) and *HLA* class II (HLA-DR, -DQ, and -DP). The encoded molecules play an important role in the modulation of immune responses and self versus non-self recognition. Japanese studies identified DRB1*09:01 and DQB1*03:03 as risk alleles for sporadic ANE (Hoshino et al., 2016), and DPB1*04:01:01 as a protective allele for AESD (Kasai et al., 2022b). The association of DQB1*03:03 and ANE was also reported in a Korean case (Oh et al., 2004). DRB1*09:01 is common in east Asia but rare in European populations, which may partially account for the high incidence of ANE in east Asia.

Interleukin-1β and its receptors constitute a critical pathway both in neuroinflammation and neuroprotection. In the promotor of the *IL1B* gene, there is an upstream variant, rs16944 (*IL1B*-511T > C), which is associated with high expression of IL-1β (Aguet et al., 2020). Previous studies in Asian countries demonstrated a positive (risk) association of rs16944 with febrile seizures (FS) (Tsai et al., 2002; Kira et al., 2005; Yu et al., 2018). By contrast, a recent study showed a negative (protective) association of rs16944 with AESD (Shibata et al., 2022). AESD has a genetic background distinct from FS, although the clinical presentation of AESD at its onset is indistinguishable from that of prolonged FS.

Interleukin 1 receptor antagonist (IL-1Ra), an endogenous antagonist of IL-1β, is encoded by the *IL1RN* gene. A variable number of tandem repeats (VNTR) polymorphism in *IL1RN* intron 2 is associated with many chronic inflammatory diseases and with FS (Tsai et al., 2002). A Japanese study found an association of a VNTR allele, RN2 (two repeats), and FIRES (Saitoh et al., 2016). Since an RN2 allele is associated with reduced *IL1RN* mRNA expression and enhanced IL-1β production (Santtila et al., 1998; Korthagen et al., 2012), RN2 may confer susceptibility to excessive neuroinflammation.

Interleukin 10 (IL-10), an anti-inflammatory cytokine, is encoded by the *IL10* gene. A genetic polymorphism in its promotor region is a combination of two SNPs, rs1800871 and rs180072, which show complete linkage and are associated with a reduced production of IL-10. Our recent study demonstrated an association of this polymorphism with ANE (Ishii and Hirose, 2018).

Toll-like receptor 3 (TLR3), a pattern recognition receptor for double stranded RNAs, activates innate immune responses upon viral infections. A loss-of-function mutation of the *TLR3* gene was found in a single case of influenza-associated encephalopathy (Hidaka et al., 2006), and in multiple cases of herpes simplex virus encephalitis (Zhang et al., 2007).

Serine/threonine kinase 39 (STK39), an activator of the p38 mitogen-activated protein kinase (MAPK) pathway, mediates cellular stress-activated signals (Johnston et al., 2000), and is involved in the pathogenesis of brain edema (Kahle et al., 2008). A GWAS study in Japan individuals has recently identified a variant in intron located in the enhancer region of the *STK39* gene as a candidate susceptibility factor of AESD (Kasai et al., 2022a).

### Genes regulating metabolism

Inherited metabolic errors of fatty acids, organic acids, carbohydrates and the urea cycle may be clinically manifested with acute encephalopathy (Mizuguchi et al., 2007). In particular, disorders of energy metabolism often show coma and/or convulsion because the brain is one of the most energy- or ATP-consuming organs in the human body. Common biochemical findings of metabolic encephalopathy include hypoglycemia, lactic acidosis, ketonuria, metabolic acidosis, hyperammonemia, hypocarnitinemia, and hepatic dysfunction (Mizuguchi et al., 2021).

Many causative genes of these metabolic disorders are listed as genetic risk factors of acute encephalopathy. Most of them are located on auto-chromosome, some on X chromosome, and only a few on mitochondrial DNA.

Carnitine palmitoyltransferase II (CPT2), a key enzyme of lipid metabolism located on the mitochondrial inner membrane, catabolizes acylcarnitine and produces acyl-CoA, which in turn is catabolized through β-oxidation to produce ATP. Homozygous mutations of the *CPT2* gene may cause a Reye-like syndrome (Vianey-Saban et al., 1993). Several polymorphisms in exon 4 and exon 5 of the *CPT2* gene causes thermolability, a severe loss of enzymatic activity at high body temperature despite a minimal or mild reduction at normal body temperature. Several studies in Japan have shown an association of the *CPT2* thermolabile polymorphism, in particular rs2229291 (F352C) in exon 4, with acute encephalopathy. Earlier studies reported its associations with severe syndromes, such as influenza-associated and other encephalopathy with fatal or poor outcome (Chen et al., 2005; Yao et al., 2008; Kubota et al., 2012), AESD and ANE (Shinohara et al., 2011), whereas later studies demonstrated those with a mild syndrome, MERS, and with entire acute encephalopathy (Shibata et al., 2019). The rs2229291 variant is common in east Asians but not reported in Caucasians (NCBI),^1^ which may account for the high incidence of acute encephalopathy in east Asia.

Ran-binding protein 2 (RANBP2), or nucleoporin 358, is a component of the nuclear pore complex involved in nucleocytoplasmic transport, pro-inflammatory signaling and mitochondrial trafficking. Missense mutations in the *RANBP2* gene have been found in ANE1 (Neilson et al., 2009; Bergamino et al., 2012; Sell et al., 2016; Hu et al., 2022). Studies on the pathogenesis of ANE1 have hypothesized the impact of *RANBP2* mutation on immune response and energy metabolism (Cho et al., 2012; Levine et al., 2020). In support of the metabolic hypothesis, a recent study demonstrated an attenuated ability of mutated RANBP2 to bind to cytochrome *c* oxidase copper chaperone, COX11 (Shibata et al., 2021), which may affect glucose metabolism because RANBP2, when bound to COX11, suppresses its inhibitory activity over hexokinase 1 (Aslanukov et al., 2006). Pathologically, bilateral symmetric brain lesions in ANE1 resembles those in Leigh syndrome, a mitochondrial encephalopathy, which may be explained by mitochondrial dysfunction common to both conditions.

### Genes regulating neuronal excitation

The α1 (Nav1.1) and α2 (Nav1.2) subunits of neuronal voltage-gated sodium channels are encoded by the *SCN1A* and *SCN2A* genes, respectively. Mutations of these genes cause various epileptic syndromes of variable clinical presentation. Dravet syndrome is a severe epilepsy characterized clinically by fever sensitivity, whose main genetic cause is missense, truncation, indel and microdeletion mutations of the *SCN1A* gene. In patients with Dravet syndrome, the incidence of acute encephalopathy is high, and acute encephalopathy is an important cause of death (Takayanagi et al., 2010; Tang et al., 2011; Okumura et al., 2012). Vaccination, particularly for pertussis, may also trigger acute encephalopathy in children having a *SCN1A* mutation (Berkovic et al., 2006). Notably, acute encephalopathy may occur in patients with a *SCN1A* mutation but without Dravet syndrome, many of whom being apparently healthy prior to the onset of encephalopathy. In a Japanese cohort of 87 patients with severe encephalopathy including ANE and AESD, but without underlying neurological disorders, three reportedly had *SCN1A* missense mutations (Saitoh et al., 2012). Another study demonstrated an association of *SCN1A* non-synonymous variants with AESD. Many of these variants were located in the transmembrane segments or pore-forming regions of the SCN1A molecule (Shibata et al., 2020).

Mutations of the *SCN2A* gene has been found in a small number of Japanese cases of acute encephalopathy including FIRES, AESD, ANE and AESD (Kobayashi et al., 2012; Fukasawa et al., 2015; Saitoh et al., 2015a). A polymorphism in *SCN2A* intron 22, rs 1864885, has shown a possible association with FIRES (Saitoh et al., 2016).

Adenosine A2A receptor (ADORA2A) enhances excitatory neurotransmitter release, and its experimental activation in the brain lowers the threshold of hyperthermia-induced seizures in rat pups (Fukuda et al., 2011). There are in the *ADORA2A* gene four SNPs, rs2298383, rs5751876, rs35320474, and rs4822492, which show complete linkage and comprise only two haplotypes (A and B) in Japanese population. A case-control association study showed that the risk of AESD is higher in AA than in AB and BB diplotypes. Since *ADORA2A* mRNA expression and cAMP production is higher in AA, this diplotype likely alters the intracellular adenosine/cAMP cascade, thereby promoting seizures and excitotoxic damage in AESD (Shinohara et al., 2013).

Voltage-dependent calcium channel a-1 (CACNA1A) is a subunit of P/Q type calcium channel distributed throughout the brain. Mutations in the *CACNA1A* gene are clinically manifested with diverse phenotypes including familial hemiplegic migraine and episodic ataxia. Several cases of migraine reportedly had hemiconvulsion-hemiplegia syndrome, an encephalopathy syndrome showing a significant overlap with AESD (Yamazaki et al., 2011; Ohmura et al., 2012).

The α3-subunit of the Na(+)/K(+) -ATPase (ATP1A3) is an electrogenic cation pump in the brain regulating concentration gradients of sodium and potassium ions across the plasma membrane. Mutations in the *ATPA1A3* gene clinically present with multiple phenotypes including alternating hemiplegia of childhood, rapid-onset dystonia parkinsonism, cerebellar ataxia, areflexia, pes cavus, optic atrophy, sensorineural hearing loss (CAPOS) syndrome and early-infantile epileptic encephalopathy. Specific *ATPA1A3* variants may cause acute encephalopathy: relapsing encephalopathy with cerebellar ataxia (Dard et al., 2015; Biela et al., 2021).

Rho-related BTB domain-containing protein 2 (RHOBTB2) is an atypical Rho GTPase and a substrate for the cullin-3-based ubiquitin ligase complex that recruits target proteins for degradation. Mutations in the *RHOBTB2* gene cause epileptic encephalopathy, which is often complicated by febrile status epilepticus followed by acute encephalopathy (Belal et al., 2018).

Protocadherin 19 (PCDH19) is a transmembrane regulating cell-cell contact and involved in neuronal proliferation, migration and synaptic function. Mutations in the *PCDH19* gene on X chromosome cause neurodevelopmental PCDH-clustering epilepsy syndrome in female hemizygotes. A female patient with a missense mutation in *PCDH19* reportedly had acute encephalopathy mimicking FIRES (Specchio et al., 2011).

Heterogeneous nuclear ribonucleoprotein U (HNPRU) is a component of spliceosome that plays a role in brain development. Mutations in the *HNPRU* gene cause early-onset epileptic encephalopathy, which is occasionally complicated by AESD (Shimada et al., 2018).

Hamartin and tuberin, encoded by *TSC1* and *TSC2*, respectively, form a complex to play a pivotal role in the mammalian target of rapamycin (mTOR) pathway. Mutations in these genes cause tuberous sclerosis complex (TSC), showing focal cortical dysplasia (cortical tubers) and epilepsy often resistant to antiepileptic drugs. Triggered by febrile infections, TSC is often complicated by febrile status epilepticus followed by acute encephalopathy, including AESD (Numoto et al., 2021). Other congenital syndromes with disorders of cerebral cortical development are occasionally complicated by AESD and other syndromes of acute encephalopathy (Hirayama et al., 2017).

### Genes involved in thermoregulation

The neurotrophic tyrosine kinase receptor 1 (NTRK1) is a neurotrophin receptor involved in neuronal survival and differentiation. Mutations in the *NTRK1* gene cause hereditary sensory and autonomic neuropathy type IV (HSAN-IV), or congenital insensitivity to pain with anhidrosis (Indo et al., 1996). Common symptoms of HSAN-IV include recurrent episodes of hyperthermia and unexplained fever, leading occasionally to acute encephalopathy resembling heatstroke (Iwanaga et al., 1996).

The α9 (Nav1.7) subunit of neuronal voltage-gated sodium channels (SCN9A) is expressed in somatic and visceral neurons and in sympathetic ganglion neurons, and plays a role in pain signaling. Mutations in the *SCN9A* gene may cause erythermalgia, a rare neuropathy characterized by attacks of burning pain in the extremities in response to warm stimuli. Hypothermia, an occasional complication of erythermalgia, reportedly lead to fungal pneumonia which in turn triggered the onset of MERS (Takahashi et al., 2007).

The α10 (Nav1.8) subunit of neuronal voltage-gated sodium channels (SCN10A) is expressed in nociceptive neurons, and is involved in pain sensation. Biallelic mutations in the *SCN10A* genes may cause epileptic encephalopathy, neuromuscular disease, bradycardia and/or anhidrosis. A patient with compound missense mutations reportedly had FIRES (Kambouris et al., 2016).

### Genes maintaining myelin sheath

Myelin regulatory factor (MYRF) is a transcription factor necessary for oligodendrocyte differentiation and the maintenance of mature oligodendrocytes and myelin structure (Emery et al., 2009; Koenning et al., 2012). A heterozygous missense mutation of the *MYRF* gene causes familial/recurrent MERS, or encephalopathy with reversible myelin vaculotion (Kurahashi et al., 2018). Except for the familial occurrence showing Mendelian, autosomal dominant inheritance, this condition is clinically indistinguishable from MERS type 2, in which intra-myelinic edema is suggested as the pathologic substrate (Takanashi, 2009). Interestingly, an X-linked demyelinating disorder, X-linked Charcot-Marie-Tooth disease type 1 (CMTX1), is occasionally complicated by familial/recurrent, transient leukoencephalopathy showing reversible, cerebral white matter and callosal lesions mimicking those of MERS type 2. CMTX1 is caused by mutations in the *GJB1* (gap junction beta 1) gene encoding another factor for myelin maintenance, connexin 32. Often triggered by a febrile infection, many male hemizygotes and several female heterozygotes reportedly had transient leukoencephalopathy, whose main clinical findings are not disturbance of consciousness, but motor and sensory signs (Hanemann et al., 2003; Sato et al., 2012; Wang and Yin, 2016).

## Conclusion

This review tried to provide a comprehensive list of risk factors of acute encephalopathy, and described many viruses, drugs and gene variants which are involved, either as initiating or aggravating factors, at the onset or during the evolution of pathogenetic process leading to brain edema. The risk factors were classified according to the main pathogenetic event in which each factor is involved. Due to the limitation inherent to this approach, however, this review failed to describe some risk factors of acute encephalopathy complicating certain neurologic disorders whose pathomechanism is complex and/or poorly understood, such as neonatal hypoxic-ischemic encephalopathy, periventricular leukomalacia, congenital cytomegalovirus infection, Prader-Willi syndrome, congenital adrenal hyperplasia and incontinentia pigmenti (Abe et al., 2011, 2016; Hirayama et al., 2017).

The complex associations of these factors with pathogenetic mechanisms have multiple aspects. First, a single risk factor may be associated with multiple syndromes of different mechanisms. For example, a thermolabile *CPT2* variant, a risk factor in energy metabolism, is associated not only with metabolic encephalopathy, but also with other syndromes such as AESD and MERS. Second, multiple risk factors of different categories may converge to develop a single syndrome. For example, common variants of three genes, *IL1B* (immune response), *CPT2* (metabolism) and *ADORA2A* (neuronal excitation), are all associated with AESD.

The complex pathomechanism of acute encephalopathy may hamper its prevention and treatment. On the other hand, every risk factor could be a candidate target for preventive measures and/or therapeutic intervention. For example, vaccines against influenza virus and rotavirus may ameliorate the incidence, mortality and morbidity of influenza- and rotavirus-associated encephalopathy, respectively. Restriction in the use of aspirin caused a dramatic decrease in classical Reye syndrome (Belay et al., 1999). Molecular target therapies against proinflammatory cytokines may improve the outcome of severe syndromes: anakinra and tocilizumab for FIRES (Aledo-Serrano et al., 2022) and tocilizumab for ANE (Koh et al., 2019). The attenuation of CPT2 in acute encephalopathy may be rescued by a lipid-lowering agent, bezafibrate (Yao et al., 2011). In conclusion, studies to explore various risk factors should suggest many candidate targets for improved prevention and treatment of acute encephalopathy in the future.

## Author contributions

MM contributed to the writing of the manuscript. AS, MK, and AH contributed to the refinement of the manuscript. All authors read and approved the submitted version.

## Footnotes

1
http://www.sanger.ac.uk
